# A high-density SNP panel reveals extensive diversity, frequent recombination and multiple recombination hotspots within the chicken major histocompatibility complex *B* region between *BG2* and *CD1A1*

**DOI:** 10.1186/s12711-015-0181-x

**Published:** 2016-01-07

**Authors:** Janet E. Fulton, Amy M. McCarron, Ashlee R. Lund, Kara N. Pinegar, Anna Wolc, Olympe Chazara, Bertrand Bed’Hom, Mark Berres, Marcia M. Miller

**Affiliations:** Hy-Line International, Dallas Center, IA USA; Iowa State University, 239C Kildee, Ames, IA 50011 USA; Department of Pathology and Centre for Trophoblast Research, University of Cambridge, Cambridge, UK; Génétique Animale et Biologie Intégrative, INRA, AgroParisTech, Université Paris-Saclay, 78350 Jouy-en-Josas, France; Department of Animal Sciences, University of Wisconsin, Madison, USA; Department of Molecular and Cellular Biology, Beckman Research Institute, City of Hope, Duarte, CA USA

## Abstract

**Background:**

The major histocompatibility complex (MHC) is present within the genomes of all jawed vertebrates. MHC genes are especially important in regulating immune responses, but even after over 80 years of research on the MHC, much remains to be learned about how it influences adaptive and innate immune responses. In most species, the MHC is highly polymorphic and polygenic. Strong and highly reproducible associations are established for chicken MHC-*B* haplotypes in a number of infectious diseases. Here, we report (1) the development of a high-density SNP (single nucleotide polymorphism) panel for MHC-*B* typing that encompasses a 209,296 bp region in which 45 MHC-*B* genes are located, (2) how this panel was used to define chicken MHC-*B* haplotypes within a large number of lines/breeds and (3) the detection of recombinants which contributes to the observed diversity.

**Methods:**

A SNP panel was developed for the MHC-*B* region between the *BG2* and *CD1A1* genes. To construct this panel, each SNP was tested in end-point read assays on more than 7500 DNA samples obtained from inbred and commercially used egg-layer lines that carry known and novel MHC-*B* haplotypes. One hundred and one SNPs were selected for the panel. Additional breeds and experimentally-derived lines, including lines that carry MHC-*B* recombinant haplotypes, were then genotyped.

**Results:**

MHC-*B* haplotypes based on SNP genotyping were consistent with the MHC-*B* haplotypes that were assigned previously in experimental lines that carry *B2*, *B5*, *B12*, *B13*, *B15*, *B19*, *B21*, and *B24* haplotypes. SNP genotyping resulted in the identification of 122 MHC-*B* haplotypes including a number of recombinant haplotypes, which indicate that crossing-over events at multiple locations within the region lead to the production of new MHC-*B* haplotypes. Furthermore, evidence of gene duplication and deletion was found.

**Conclusions:**

The chicken MHC-*B* region is highly polymorphic across the surveyed 209-kb region that contains 45 genes. Our results expand the number of identified haplotypes and provide insights into the contribution of recombination events to MHC-*B* diversity including the identification of recombination hotspots and an estimation of recombination frequency.

**Electronic supplementary material:**

The online version of this article (doi:10.1186/s12711-015-0181-x) contains supplementary material, which is available to authorized users.

## Background

The major histocompatibility complex (MHC) is a polymorphic gene region that is conserved in all jawed vertebrates. The MHC is characterized by the presence of a number of conserved genes that have remained together during evolution. In chickens, MHC genes are divided into two large gene clusters, MHC-*B* and MHC-*Y*, which are located in separate haplotypes but on the same chromosome [1]. Many of these genes contribute to immune responses with specific alleles at some loci that potentially play a major role in the genetic mechanisms of resistance to infectious diseases. The chicken B blood-group system [2], now referred to as the MHC-*B* region, is an important component for disease resistance in chickens. Links between particular MHC-*B* haplotypes and resistance to infectious diseases in chickens led to early and extensive investigations into the diversity of chicken MHC-*B* haplotypes. Although many genes within MHC-*B* are polymorphic and polygenic, to date, most associations between MHC-*B* and resistance to diseases are defined only at the level of the haplotype and the roles of only a very few individual loci are documented. For example, the classical MHC class I *BF2* locus was shown to be involved in the resistance to Rous sarcoma virus challenge (defined as tumor regression over a time-course during which an adaptive immune response is likely to occur). It was demonstrated that immunization of chickens with a single v-*src* peptide that was predicted to bind to the MHC class I BF2*12 binding groove was sufficient to induce specific tumor regression [3]. Another study that showed that the *BG1* locus had a highly significant influence on the occurrence of Marek’s disease [4] provided evidence that genes other than those involved in classical peptide antigen presentation also contribute to MHC-*B*-linked disease resistance. To what extent genes in the MHC-*Y* cluster, which was more recently discovered, contribute to disease resistance, is less well defined and is currently under investigation [5]. To understand the genetic basis of disease resistance, more powerful methods are needed to investigate the genetic variability of the highly polymorphic genes within the chicken MHC.

The genetic contributions of chicken MHC-*B* haplotypes to disease resistance are especially important for several agriculturally significant avian diseases that are caused by viruses, bacteria and eukaryotic parasites [4, 6–17] and for the efficacy of vaccination [18–22]. Several inbred MHC-*B* lines, including congenic lines, were developed mostly within the White Leghorn breed to investigate the link between MHC-*B* haplotypes and resistance to infectious diseases [23–28]. MHC-*B* haplotypes were originally identified by using alloantisera in hemagglutination assays and thus, haplotypes that include the *BG* and *BF* loci could be followed and selected for in fully pedigreed families [29, 30]. In 1982, an international exchange of alloantisera and blood samples led to the standardization of 27 serologically-defined MHC-*B* haplotypes [29]. Many of these haplotypes form the basis for experimental investigations on the function and structure of the MHC-*B* region. In 2004, limited sequence data for the chicken MHC supported the definition of these 27 standard haplotypes [31]. More recently, additional and more extensive sequencing of a 59-kb region of the MHC-*B* confirmed the differences between 14 standard haplotypes across this region and provided evidence that mutation, recombination and gene conversion events have contributed to the diversity of MHC-*B* haplotypes [32].

The extent of the diversity of MHC-*B* haplotypes across chicken breeds and strains is unknown largely because of the lack of readily-applied methods. Early work was limited by the fact that *B* alloantisera are rarely haplotype-specific and unpredictable cross-reactivity made it difficult to identify MHC-*B* haplotypes across breeds and lines. The poorly defined basis of alloimmune responses restricted the application of serological typing to defined lines and breeds that contained only a few haplotypes [29, 30]. Many of the MHC-*B* haplotypes of non-domesticated chickens and other breeds, including those used for the production of brown shell eggs and for meat (broiler), remained poorly defined.

More recently, several DNA-based MHC-*B* typing assays have been developed to detect and study differences within chicken MHC haplotypes between experimental and commercial lines. For example, methods based on Southern hybridization for several members of the same gene family, single strand conformation polymorphism (SSCP) methods to define alleles at individual loci, typing methods based on the complex variable number tandem repeat (VNTR) LEI0258, and single nucleotide polymorphism (SNP) assays across limited regions have been effective in identifying MHC variation in experimental, commercial and numerous indigenous breeds [33–49]. Among these, the typing method based on the VNTR LEI0258 considerably simplified and enhanced the capacity to assign MHC-*B* haplotypes [21, 46, 50].

Currently, typing based on the VNTR LEI0258 [52] is the most used method to obtain genetic information on the MHC-*B* region. An analysis of 35 previously serologically-defined MHC-*B* haplotypes showed that LEI0258 alleles associate well with MHC-*B* haplotypes [46]. LEI0258 alleles vary in length because they include variable numbers of unusual 13- and 12-bp long tandem repeats and also SNPs and small indels within the flanking sequences [46]. Since length variations of the LEI0258 alleles are large and often discrete, many alleles can be distinguished by agarose gel electrophoresis. LEI0258 typing has been applied to detect MHC-*B* differences in multiple populations in which the MHC-*B* was not previously defined [14, 17, 53–59] and such studies on indigenous strains of chickens have linked helminth or bacterial infections with the MHC-*B*, thus establishing that MHC-*B* is involved in the resistance to pathogens of non-laboratory selected lines [15, 17, 60].

Although highly valuable, it is clear that LEI0258 typing alone is not sufficient to fully define the variability of the MHC-*B* region. Indeed, MHC-*B* haplotypes, such as the *B2* and *B15* haplotypes that differ based on serology data and genomic sequence, have the same LEI0258 allele size [46]. In addition, since LEI0258 typing defines only a single location, it is not useful for the identification of recombinant haplotypes, which are inherent to the MHC-*B* region [26, 32, 61–68].

The strong relationship between MHC-*B* variability and resistance to disease and the deficiencies of the detection methods that are currently used warrant the development of a reliable MHC-*B* detection method. With the goal of developing a means for high-resolution MHC-*B* typing across multiple breeds and populations, we have identified and tested 101 SNPs across the 209,296-bp MHC-*B* reference region (GenBank accession number: AB268588) that was reported by Shiina et al. [69] and that contains 45 genes, which are nearly all involved in innate and adaptive immunity. We tested the method with thousands of samples and detected structural similarities and differences among haplotypes, identified new recombinant haplotypes, and developed a reliable and rapid means for MHC-*B* typing.

## Methods

### Genetic material

Genotyped DNA samples included a subset of the samples that had previously been typed using the VNTR LEI0258 [46]. These samples were mostly from research and commercial lines for which MHC-*B* haplotypes are serologically defined [46] and included samples from homozygous individuals of inbred or selected lines that represented well-identified MHC-*B* types [provided by ME Delany (University of California Davis lines), WE Briles (Northern Illinois University pedigreed families), RL Taylor Jr. (University of New Hampshire congenic lines), HD Hunt (USDA/ADOL lines) and SJ Lamont (Iowa State University lines)]. Each haplotype from each source was represented by at least two different individuals. In addition, extensive typing was carried out on multiple samples from many elite layer lines that are used in commercial production of both white shell and brown shell eggs. In addition, all the males of one generation of each elite population were genotyped as well as several males from other generations that carry rare and novel LEI0258 variants. Complete genotyping and LEI0258 typing information was obtained for over 5000 samples. An additional 1351 samples were obtained from 17 heritage chicken lines that are maintained at various universities in Canada and the USA [70]. DNA from the same RJF Line 256 individual used as the reference for the chicken genome sequence project (provided by H. Cheng, USDA) was also tested with this SNP panel.

In addition to the defined standard and recombinant lines, a population of 1189 fully pedigreed commercial (i.e., multiple line hybrids) egg laying birds produced from the mating of 15 males with 142 females was genotyped. Sires, dams and progeny were genotyped with a sub-set of SNPs that are known to define the haplotypes expected in the population. The birds whose haplotypes failed to conform to the expected parental haplotypes were subsequently genotyped with the full set of 101 SNPs.

### LEI0258 typing

Representative samples of each defined haplotype were typed for LEI0258 using the Beckman CEQ 8800 instrument (Beckman-Coulter, Fullerton, CA, USA), according to the protocol in [46]. Since the size of the observed allele can vary slightly depending on the detection platform used, all allele sizes were converted to their equivalent when using the ABI 377 (Applied Biosystems, Foster City, CA, USA) platform as reported in [46].

### SNP panels

The SNP panel was created in several steps starting with a set of 96 SNPs that was developed for the Illumina Golden Gate assay for MHC-*B* [71]. To improve accuracy and reduce the costs associated with the original Illumina Golden Gate assay [51], SNP detection was modified to a more scalable PCR-based (KASP™ genotyping) assay [72]. This method identifies alleles by using one fluorescent label for each allele i.e., VIC™ and FAM. Genotype information is obtained based on the relative amount of each fluorescent dye detected, with intermediate levels of both labels detected for the heterozygotes. We used the genotyping software Kraken (LGC, Middlesex, UK) to determine genotypes and analyze the data. Several of the 96 SNPs from the original GoldenGate genotyping assay did not perform well, i.e. either they failed to provide any genotype information or they indicated heterozygosity for samples from individuals that were known to be homozygous for the MHC; in these cases, the SNPs were replaced with adjacent SNPs. SNPs were considered as reliable and thus included on the final panel only if both alleles were consistently detected and genotyping results were uniform in tests with well-defined samples. Samples from individuals that were known to be homozygous for the MHC had to be consistently homozygous for the same allele, while those from pedigreed individuals that were known to be heterozygous had to have alleles that were consistent with those present within the parental haplotypes. The initial panel of SNPs covered the region between 30,189 and 240,933 bp (GenBank accession number: AB268588), i.e. between the *BZFP3* and *CD1A1* genes.

**Fig. 1:**
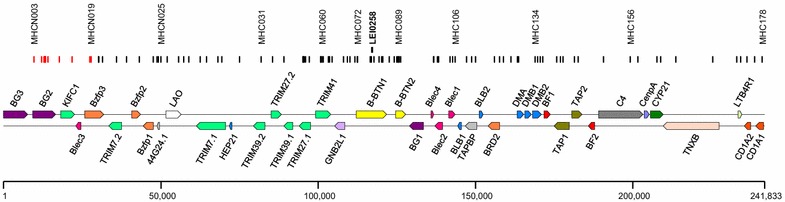
Gene map of the MHC-*B* region based on [GenBank: AB268588]. Positions of SNPs in the high-density panel and of the LEI0258 marker (above the SNPs) are shown. Only those SNPs indicated in *black* were used for the definition of the MHC-*B* haplotype. SNPs indicated in *red* frequently provided evidence of gene duplication or deletion. The scale unit is bp

Later, an additional 18 SNPs were included to extend the SNP coverage of the MHC-*B* region and to improve SNP density within the first quarter of this region. These “MHCNew SNP” included SNPs that were present in the first 30,000 bp of the MHC-*B* sequence that contains the *BG2*, *KIFC1*, and *BLEC3* genes (GenBank accession number: AB268588), and an additional SNP in the *BZFP3* gene (see Additional file 1: Table S1). The additional SNPs that were localized upstream of the region used for the original SNP panel are indicated by red lines in Fig. 1. SNPs for this region were identified by aligning the overlapping regions between the two following sequences GenBank accession numbers AB268588 and KC955130 (that contain the *BG2*, *KIFC1* and *BLEC* genes in haplotype *B12*). SNPs that were within introns were selected in an attempt to bias the selection towards gene-specific SNPs. Only SNPs with flanking sequences showing single matches by BLAST search against the sequence GenBank accession number: AB268588 were included, which resulted in the identification of seven SNPs for the region between 9551 and 13,992 bp. Eleven SNPs within the region between 17,607 and 62,274 bp in the sequence GenBank accession number: AB268588 were added to provide supplementary genotype information to better define recombinant MHC-*B* haplotypes. These SNP variants were identified from resequencing data of Hy-Line elite lines [73]. The flanking sequences for these 11 SNPs were BLAST-searched against the RJF reference genome (galGal4.0), which revealed that SNP MHCNew14 was present at two locations with two reference SNP (rs) numbers. SNPs MHCNew18 and MHCNew19 were each present in both the galGal4.0 sequence that was assigned to chromosome 16 and in the chromosome Unassigned sequence.

One hundred and one SNPs were included in the final panel (see Additional file 1: Table S1). Additional file 1: Table S1 provides detailed information on each SNP (name, bp and gene locations in the sequence GenBank accession number: AB268588, and the NCBI rs numbers when available), the sequence of both the common and allele specific primers and, the Red Jungle fowl SNP followed by the expected SNP variant. Optimal primer design used either the forward or reverse strand and this is also indicated (see Additional file 1: Table S1).

### Haplotype identification

The MHC SNP panel contains 101 SNPs, but only the 90 SNPs that encompass the region between 30,189 and 240,933 bp (i.e. between SNPs MHCJ06 and MHC178) were used to define the haplotypes due to the complex results seen with the first 11 SNPs (see Additional file 2: Table S2). Haplotypes were identified in a three-step analysis. First, DNA samples from birds that were homozygous for MHC-*B* were used to define individual SNP-based patterns (haplotypes). Second, the SNP patterns from MHC-*B* heterozygous birds were visually compared to the SNP patterns provided by homozygous birds in the first step. The SNP patterns for defined haplotypes were subtracted and the remaining SNP patterns were used to define the other SNP haplotypes present in the heterozygous birds. This analysis was performed independently within each line in which MHC-*B* was segregating. Finally, independently-defined haplotypes were compared across lines to identify common haplotypes.

### Identification of clusters of SNP haplotypes

Independently-defined SNP haplotypes were compared across lines to determine the degree of genetic similarity, and haplotypes were clustered using computational phylogenetic methods. First, the R software package APE [74] was used to compute the pair-wise uncorrected fractional dissimilarity among all SNP haplotypes. Second, neighbor-joining (NJ) topology [75] was constructed using the computed genetic distances to depict clusters of SNP haplotypes. Third, statistical support for each topological branch was derived from a majority-rule consensus [76] of 1000 bootstrap replicates of the original haplotype data [77]. Each bootstrap value indicates, as a percentage, the occurrence of a branch found in a majority (>50 %) of the randomized haplotype data. Haplotypes were grouped into clusters using the method of Prosperi et al. [78]. In our analysis, this model groups haplotypes into clusters if the median pair-wise patristic distance (MPPD) between each node of a putative cluster of haplotypes is below a threshold. Two implementations of this model were used. Using the NJ topology, we first computed the MPPD of all nodes (internal and terminal) in the topology. Next, we computed a quantile distribution of these distances to determine a suitable similarity threshold. Specifically, a cluster is defined if the distribution of its MPPD is below a percentile of the MPPD of the entire topology. We set this percentile to 10 %. In other words, cluster membership was restricted to the grouping of haplotypes that were more closely related to each other than those from the randomly selected 90 % haplotypes. We also evaluated each cluster in terms of bootstrap reliability. With this complementary constraint, only haplotypes that met the 10 % percentile MPPD threshold and exhibited a bootstrap reliability greater or equal to 70 % were considered to form a cluster. The resulting clusters that comprised two or more haplotypes and nine examples of distinct single haplotypes were subsequently assigned an alphabet letter name.

### Naming of SNP haplotypes

SNP haplotypes that were defined from the set of 90 SNPs that lie between SNPs MHC0J6 and MHC178 were named “BSNP” followed by a letter that defined to which cluster the haplotype belonged and then by a two digit number to distinguish the BSNP from other members of the same cluster. If the size of the LEI0258 allele was known, it was indicated within parentheses. For example, the first haplotype (see Additional file 2: Table S2) is BSNP-A01(345). If the serological B haplotype was known, it was given following the LE0258 allele size, as exemplified in the second and third members of the “A” cluster, BSNP-A02 (357;B75) and BSNP-A03 (369;B21.1). If a BSNP haplotype had multiple LEI0258 alleles, the additional LEI0258 alleles were also included, i.e., BSNP-A09A (357,369;BQ). If the same BSNP pattern was found for lines that were previously defined as serologically different, this serological information was included i.e., BSNP-B03 (249;B15.2,B22) and BSNP-J04 (539,552;B19,B19.1). In the four cases for which the same BSNP was associated with a polymorphism in the first 30,000 bp, an additional letter was added to the name (see Additional file 2: Table S2), i.e. BSNP-A09A (357,369;BQ) and BSNP-A09B (357). The BSNP haplotype of the individual that was sequenced for the chicken genome project was also determined and the letters “RJF” were then added, i.e. BSNP-A07RJF*(369).

### Naming of recombinant SNP haplotypes

The recombinant SNP haplotypes were named using a numerical designation that followed the order in which they were identified (i.e., BSNP-Rec01, BSNP-Rec02, etc.), (see Additional file 3: Table S3). Additional information, such as the size of LEI0258 alleles, and previously accepted recombinant names were included when available (see [31]), for example, BSNP-Rec01 (309; B2R1, B2R3). Novel recombinant haplotypes were scored as recombinant only if both parental haplotypes were also identified within the line that carried the recombinant haplotype.

### Quantitative PCR

We performed quantitative PCR to examine genome copy number for the region surrounding the MHCNew10 SNP. We used the following primers: MHCNew10F, TGCAAGTTAAATGCTGCAAATC and MHCNew10R, AAATGCTTGGTGAGAAGCG with LNA probe CT+G+TA+CA+CA+GAA+GG (IDT, Iowa City IA, USA). The DNA of each sample was amplified in six replicates using a common master mix (Quantifast Probe PCR+, Qiagen, Germantown, MD, USA). DNA was diluted to 20 ng/µL and 3 µL were used for each reaction. An assay for a single copy number gene *RNAseP* was included in each well as an internal control. Additional controls included DNA from individuals known to be trisomic or tetrasomic for the MHC (MHC-*B15*) [79]. Results were normalized against the internal control and then against a disomic MHC-*B15* to obtain relative genome equivalents.

## Results and discussion

### Analysis of SNP haplotypes

The typing panel consisted of 101 SNPs that yielded reliable SNP genotype information across the MHC-*B* region for multiple MHC haplotypes. The 231,382-bp DNA segment (GenBank accession number: AB268588) that ranged from 9551 to 240,933 bp encompassed a region between genes *BG2* and *CD1A1*. As illustrated in Fig. 1, where each 11th SNP is indicated, SNPs were not evenly distributed across the MHC-*B* region with some parts sparsely and others more densely covered. This is due in part to the difficulty in obtaining consistent information for multiple haplotypes with some of the primer pairs used (which were excluded from the SNP panel). Many of the sparsely covered regions reflect the presence of polymorphic or repeated sequences to which SNP primers fail to hybridize uniformly.

Because the SNP data for the first ~30,000 bp of the MHC-*B* sequence (GenBank accession number: AB268588) led to complex results for several haplotypes (see Additional file 2: Table S2), genotype information for the first 11 SNPs was not used to define the haplotypes. The remaining 90 SNPs that covered the majority of the MHC-*B* sequence (GenBank accession number: AB268588) between SNPs MHC0J6 (30,189 bp) and MHC178 (240,933 bp) are indicated by black bars in Fig. 1 and the first 11 SNPs that are located between MHCNew003 (9551 bp) and MHCNew0019 (27,791 bp) are indicated by red bars. Genotype information is provided for all 101 SNPs for the MHC-*B* haplotypes that were identified for the serologically-defined lines, commercial elite layer stocks and heritage breeds (see Additional file 2: Table S2). Seventy-eight SNP haplotypes were defined from over 7500 samples.

Analysis of the data showed that this SNP panel was sufficient to identify previously defined haplotypes, differentiate new haplotypes, detect known and new recombinant haplotypes that were the result of multiple points of crossover, and to reveal special genetic features of the 5′ *BG* region. The strict conditions that were applied for inclusion of each SNP in the panel strengthen the validity of this panel for the detection of MHC polymorphism when applied in additional populations. It is likely that many additional MHC-*B* haplotypes will be identified when the panel is used to analyze other chicken populations, i.e. other defined breeds, wild populations and indigenous chicken varieties. In a separate study using this SNP panel, we identified 310 new MHC-*B* haplotypes among 199 birds from four wild red jungle fowl populations [80]. While this panel was very useful to reveal the diversity of MHC-*B* sequences, it is likely that additional sequence diversity may exist within the region covered by the panel. Just as for serologically-defined MHC-*B* haplotypes [81], it continues to be true that the identities defined by this SNP panel were presumed and further analyses (including sequencing) may disclose differences that are not found with this method.

The SNP patterns for 78 haplotypes are summarized in Additional file 2: Table S2 which also includes information on the breed(s) within which each haplotype was found. Each haplotype is named as described above with a common alphabetic designation (A to V) for the family (cluster) within which individual haplotypes are more similar than 90 % of the haplotypes of the entire population as defined by MPPD in the NJ topology. In a few cases where a SNP allele was missing, the allele was scored as F for fail.

SNP analysis of samples from lines that carry the standard serologically-defined MHC-*B* haplotypes, many of which were maintained at different laboratory locations, and from commercial breeding populations, provided SNP patterns that were consistent with the serological assignments and with the LEI0258 types defined previously [46]. For example, the SNP haplotype BSNP-K03 (261; B2, B29) was found in the *B2* standard maintained at ADOL, UCD, Hy-Line and NIU. Similarly, SNP haplotypes were also consistent for other serologically-defined haplotypes that were identified in genetic stocks from multiple sources including *B12*, *B13*, *B15*, *B19*, *B21* and *B24*. Overall, the SNP patterns were consistent with haplotypes that were previously assigned. These consistencies validate the application of this SNP panel for MHC-*B* genotyping.

Among other BSNP haplotypes, some were nearly identical except for differences at one or the other end of the 230,000 bp region, which suggests that they might result from recombination events. For example, haplotypes BSNP-O02 (309; B24) and BSNP-O03 (309; B10, B26, B76) differed by only two SNPs near the 3′-end of the MHC-*B* sequence (GenBank accession number: AB268588) (MHC157 and MHC169). Similarly, haplotypes BSNP-A09A (357, 369; BQ) and BSNP-A09B (357) differed for seven of the eight SNPs that are located near the 5′-end of the MHC-*B* sequence (GenBank accession number: AB268588) between positions 12,062–21,784 bp.

In some cases, the SNP data clarified the relationship between serologically-defined haplotypes. For example, the two serologically-defined haplotypes *B2* and *B29* (both from WL) were identical with both SNP and LEI0258 typing [BSNP-K03 (261;B2,B29)]. Similarly, the serologically-defined haplotypes *B10* from WL, *B26* from NH, and *B76* from WPR shared the same BSNP type [BSNP-O03 (309; B10, B26, B76)]. The limited availability of full comparative serological reagent panels and control blood samples used in past studies may have resulted in the unnecessary naming of new haplotypes during independent investigations. Indeed, *B29* and *B26* haplotypes may be identical to their respective counterparts *B2* and *B10*. Alternatively, the serological assignments made in earlier studies could reflect genetic differences that were detectable by using serological reagents but not by the SNP panel.

The data obtained with the SNP panel are in agreement with genome sequences described by Hosomichi et al. for 14 standard MHC-*B* haplotypes [32]. The SNPs patterns in BSNP-U01 (295; B5) and BSNP-V06 (405; B8) differed significantly between the 5′-end and *BRD2* but much less between *BRD2* and the 3′-end of the region analyzed. Similar results were found for *B12* and *B19* SNPs. The sequences of *B12* and *B19* are nearly identical between *BG1* and *TAP1* [32]. SNP genotypes are identical across the region between SNPs MHC087 (124,920 bp) and MHC144 (177,699 bp) as reported by Hosomichi et al. [32]. However, the sequences of these two haplotypes diverge in the upstream region with MHC056 (97,054 bp) being the first SNP to differ [see BSNP-J06 (461, 474, 487; B12, B12.3, B71) and BSNP-J04 (539, 552; B19, B19.1)].

### Genetic distance among MHC-*B* haplotypes

**Fig. 2:**
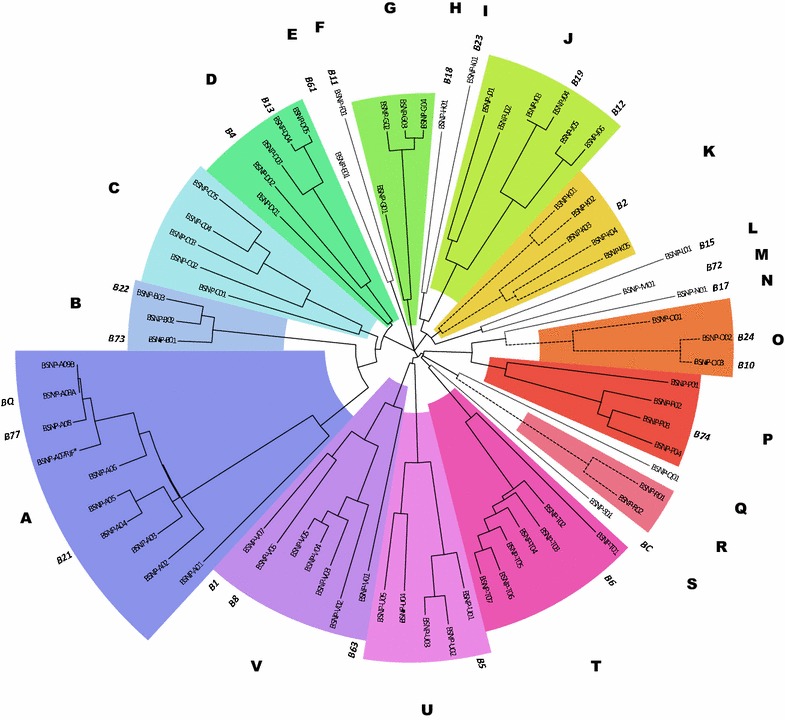
Topological clustering of MHC-*B* haplotypes based on SNP identity. The network depicts a neighbor-joining topology computed from pair-wise uncorrected fractional dissimilarity. Clusters are based on (1) the median pairwise patristic distance (MPPD) between each node with a similarity of 90 % or more (*colored clusters*) and (2) bootstrap reliability greater than 70 % (*colored clusters with solid lines*). Haplotypes that met the first but not the second, criterion are indicated by *dashed lines*. Unique haplotypes are indicated by *stippled lines*. Serological information (MHC-*B* type) is provided when available. The *outermost circle* of letters (*A* to *V*) refers to a specific cluster and is used throughout the text

To gain insights into the similarities among MHC-*B* haplotypes and to provide a logical basis for naming groups of BSNP haplotypes, we adopted methods that are typically applied to gene sequences in phylogenetic studies. The topological network in Fig. 2 illustrates the extensive diversity present in the MHC-*B* region. Based on the first constraint that haplotypes with 90 % or more MPPD similarity are part of a common group or family, the 78 SNP haplotypes were grouped into 22 families (BSNP families A through V). Most families contain multiple haplotypes. However, nine families are currently represented by a single haplotype (e.g., BSNP-S01) and in Fig. 2 they are indicated with stippled lines. To gain insight into the stability of the relationships between SNP haplotypes, we applied an additional constraint i.e. bootstrap reliability should be greater than 70 %. Three clusters (K, O, and R; branches with dashed lines) did not meet these combined criteria, which suggested enhanced differentiation among haplotypes in these clusters. Although it is likely that reticulate events in the MHC-*B* region affect all clusters of haplotypes to some degree, the prevalence of recombination or gene duplication and loss may be increased for these three clusters. We also modeled numerous haplotype networks using data in which BSNP haplotypes were variously included and excluded (jackknife resampling; data not shown). Even when analyzed with different topology reconstruction methods like maximum parsimony and maximum likelihood, the network in Fig. 2 remained strongly supported, which suggests that a majority of the SNP haplotypes that will be identified in the future will cluster within the families defined with the BSNP in this study.

In addition to defining 22 BSNP clusters, the network revealed greater separation (genetic distance) between cluster A and other clusters (Fig. 2). Multiple haplotypes within this cluster are known to confer resistance to disease including BSNP-A04(B21), BSNP-A03 (B21.1) which is serologically similar to BSNP-A04 (B21), BSNP-A05 (B5.1) which is present in the highly disease-resistant Fayoumi breed, and BSNP-A09 (BQ) that is derived from red jungle fowl. This cluster also includes haplotype BSNP-A07RJF*, which was identified in the individual that was sequenced for the chicken genome project. Those SNPs that separate the BSNP-A cluster from the other BSNP clusters are distributed across the haplotype that ranges from MHC031 to MHC178 and cover a large number of genes within the region. Haplotype BSNP-A01 separates more clearly from the rest of this cluster, as shown in Fig. 2. However, the similarities within the remaining BSNP-A members suggest that they could be recently derived.

Agglomerative clustering can capture the degree of relatedness among individual haplotypes by grouping those that are more closely related to each other. However, defining a meaningful method to create broader-scale clusters remains challenging and continues to be an open area of study. We chose an objective approach that uses a topological network combined with genetic distance and bootstrap reliability between individual haplotypes to define clusters. We show that this method is flexible and statistically robust and that it will be able to accommodate the addition of new haplotypes to the already large number of this current collection.

### MHC-*B* recombinant haplotypes

In addition to testing the SNP panel for its capacity to reveal standard and new MHC-*B* haplotypes, we assessed its ability to define recombinant MHC-*B* haplotypes and to localize crossover breakpoints. DNA available for 11 of the previously characterized recombinant haplotypes was genotyped using this SNP panel (see Additional file 3: Table S3). The parental haplotypes from which these recombinants were presumed to derive were confirmed with expected SNP allele matches and the crossover breakpoints were localized. As is often the case, the crossover breakpoints occurred within regions of sequence identical between the two parental haplotypes, thus precise breakpoints were rarely identified. For example, SNP typing for the recombinant haplotype *B2R4*, named following the conventions adopted in 2004 [31], identified BSNP-Rec03 (261; B2R4) as being composed of segments of the haplotypes BSNP-I01 (357; B23) and BSNP-K03 (261; B2, B29). Consistent with the published serological assignment of G23-L2-F2, SNP typing mapped the recombination breakpoint in a region upstream of the MHC-*B* class II genes between *TRIM7.1* and *TRIM27.2* as identified by SNPs MHC018 (64,656 bp) and MHC033 (85,359 bp). Only one pair of known recombinant haplotypes (B2R1 and B2R3 that were initially detected by using a fully pedigreed family across several generations and were considered to probably have different recombination breakpoints) failed to be distinguished by the SNP panel. These two recombinant haplotypes were identical and defined as BSNP-Rec01 (309; B2R1, B2R3). This contrasts with evidence from sequencing data that showed that *B2R1* (aka *BR2*) and *B2R3* (aka *BR4*) differed by the presence of a 225-bp indel in *B2R3* [4]. The identical SNPs that are present in *B2R1* and *B2R3* provide an example of how genetic differences may be missed with the current SNP panel. However, with the availability of sequence data, an additional KASP-based detection assay could be developed to distinguish these two recombinants and reveal the additional variability within this crossover region.

Close examination of the populations of heritage and commercial breeds in which MHC haplotypes were segregating revealed BSNP patterns that were consistent with recombination [70]. We found evidence for an additional 33 novel recombinant haplotypes, BSNP-Rec12 to BSNP-Rec44 (see Additional file 3: Table S3). For candidate recombinant haplotypes to be included, it was necessary that both parental haplotypes be present within the same population in which the recombinants haplotypes were found. The BSNP haplotype for each recombinant haplotype is listed along with the parental haplotypes from which they probably arose. BSNP-Rec20 has an apparent mismatch for SNP MHCNew28, since the allele seen for the recombinant is not found in either parental haplotype. BSNP-Rec41 and its parental haplotype BSNP-T04 (443) both failed for SNP MHC065. These two discrepancies suggest mutation and deletion events, respectively, which confirms that both mechanisms influence the evolution of the MHC as reported by Hosomichi et al. [32].

It should also be noted that there were numerous examples of apparent recombination. These were observed for individuals for which a segment of their SNP genotype was homozygous, followed by a segment that was heterozygous. One haplotype could be identified, but the second haplotype did not match any existing haplotype within the population, thus both parental alleles were not available. This precluded the accurate identification of the recombination region and did not fit the criteria used here to define recombinants, which included the presence of both parental alleles in the population.

### Recombination rates and recombination hotspots within MHC-*B*

To estimate rates of recombination within MHC-*B*, we used the haplotype data from a multiple line cross population. As above, candidate recombinant haplotypes were included only if the haplotypes from which they could be derived were present in the population. We found evidence for seven novel recombinant haplotypes among 1189 birds that were subsequently named BSNP-Rec25 to BSNP-Rec31. Each one was derived from different parental haplotype combinations and/or possessed different recombination breakpoints. The detection of seven crossovers among 1189 birds suggests a recombination rate of 0.6 per 100. This contrasts with previously reported estimates that were much lower and derived from experimental crosses typed with serological reagents suggesting 0.05 recombinant events per 100 [65, 67].

**Fig. 3:**
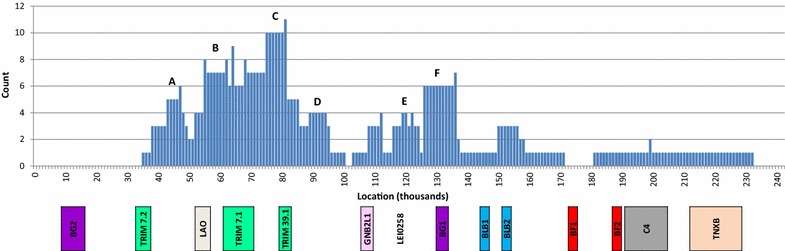
Regions of recombination for 44 unique MHC recombinants and recombination hotspots. The number of times each 1000-bp segment of the MHC was contained within the putative recombination region of 44 unique recombinants is plotted across the MHC sequence. Regions that were involved in at least four unique recombination events are suggestive of recombination hotspots and are indicated by *letters* *A*–*F*

The SNP panel data also allowed crossover breakpoints to be localized. The information on recombinant haplotypes (see Additional file 3: Table S3) includes the SNPs that flank the apparent recombination region (i.e. the region for which both parental haplotypes were identical). The number of times a particular 1000-bp region is encompassed by a recombination event was summed on the Y axis of Fig. 3. When breakpoints are mapped across the region, it is apparent that they occur at many points along the MHC. However, these crossover events are more frequent within some regions than within others. Crossovers occurred four or more times at six points along the sequence, which provides evidence for recombination “hotspots” (identified as A to F in Fig. 3). Recombination at hotspot C (between *TRIM7.1* and *TRIM39.1*) is especially frequent within the broader region between 35 and 100 kb where crossovers are frequent. Region F is another distinct hotspot, which is associated with *BG1*. Consistent with earlier findings [67], recombination seems to be less frequent in the MHC class I (*BF1* and *BF2*) and MHC class II (*BLB1* and *BLB2*) regions. However these data suggest that recombination does occur among the loci for antigen presentation and thus provides new combinations of alleles. This was also reported in a recent study on wild Red Jungle Fowl by Nguyen-Phuc et al. [80] who found the same hotspots A, E and F and one additional hotspot between *BLB2* and *BF1* using a different methodology to identify recombinant events.

### SNP genotyping in the first 30,000 bp region

In contrast to the highly consistent SNP typing for the 3′-region of the MHC-*B* region, SNPs within the first 30,000 bp frequently led to unexpected results. Two interesting phenomena were observed. First, for some haplotypes, SNP primers that were generally reliable consistently failed to provide a genotype (indicated by “-“) (see Additional file 2: Table S2, Additional file 3: Table S3). Second, for other haplotypes, the same primer pairs gave results that indicated the presence of both alleles. This occurred with a number of samples from well-characterized MHC-*B* homozygous and inbred animals. The IUPAC codes K, M, R and Y are used in the tables (see Additional file 2: Table S2, Additional file 3: Table S3) to report these results. These aberrant results were consistently found for individuals of the same serologically-defined haplotype from multiple sources and from inbred birds of different genetic backgrounds. In particular, the six SNPs MHCNew07 to MHCNew12 either consistently failed [for 15 haplotypes, e.g., BSNP-A01 (345)], or consistently provided scores for both alleles usually for four or five of the six SNPs. For example, haplotype BSNP-A04 (357; B21) consistently showed both alleles for SNPs MHCNew08, 10, 11 and 12.

**Fig. 4:**
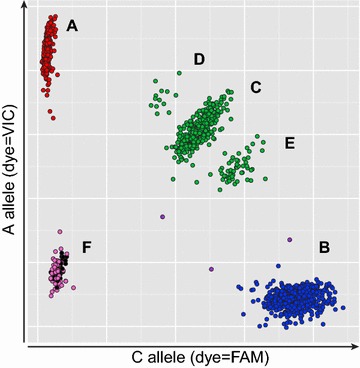
Two-dimensional diagram of fluorescence-based SNP detection with five distinct SNP clusters for SNP MHCNew10. Genotypes within each cluster are as follow: cluster *A* = A/A; cluster *B* = C/C; cluster *C* = A/C; cluster *D* = A/A/C; cluster *E* = A/C/C; cluster *F* = no template control (*black*), failed PCR (*pink*); not defined (*magenta*)

SNP MHCNew10 was selected to further investigate this region since it repeatedly showed unusual results with the KASP-SNP detection chemistry. Genotype results based on relative fluorescent values of the two allele-specific dyes can be visualized on a two-dimensional graph. The genotyping results for SNP MHCNew10 are shown on the two-dimensional cluster diagram in Fig. 4. Allele A was labeled with VIC™ and is plotted on the Y axis. Allele C was labeled with FAM and is plotted on the X axis. Normally, the two alleles would be expected to provide three well-separated SNP genotype clusters: cluster A (red in Fig. 4) with all individuals homozygous for the A allele; cluster B (blue in Fig. 4) with all individuals homozygous for the C allele; and cluster C (green in Fig. 4) with all A/C heterozygous individuals. The negative controls (i.e. no template) would be in cluster F at the origin (black in Fig. 4). Reactions that fail are expected to be rare and these clustered with the “no template” control (shown by pink circles). A few aberrant products are in magenta in Fig. 4. In a typical SNP assay in which the SNP is segregating normally, only clusters A, B, C and F and possibly a few failed and aberrant reactions would be seen.

The SNP MHCNew10 pattern in Fig. 4 is more complex. There are two additional well-separated clusters (clusters D and E in Fig. 4). The large number of failed reactions (pink) near the origin in Fig. 3 provides further evidence for the aberrant behavior of SNP MHCNew10. This “negative” result was consistently observed with specific haplotypes and included all individuals that were homozygous for those haplotypes indicated as “-” for this SNP (see Additional file 2: Table S2). In addition, all BSNP-A04 (357; B21) homozygous samples fell within cluster C, which indicates that the SNP primers detected both A and C alleles at equal levels for all BSNP-A04 (357; B21) homozygotes. Samples that were heterozygous for BSNP-A04 (357; B21) and a haplotype which had the MHCNew10 allele A fell within cluster D and similarly, samples that were heterozygous for [BSNP-A04 (357; B21)] and a haplotype with the MHCNew10 allele C fell within cluster E. This would be expected if the heterozygous individuals carried two alleles A and one allele C (for cluster D) or two alleles C and one allele A (for cluster E). These unexpected SNP results suggest that the region between SNPs MHCNew07 and MHCNew12 was absent from some haplotypes (no amplification) and was variously duplicated in other haplotypes so that extra copies of both alleles were present in some samples, and extra copies of alleles A or C were present in other samples.

**Fig. 5:**
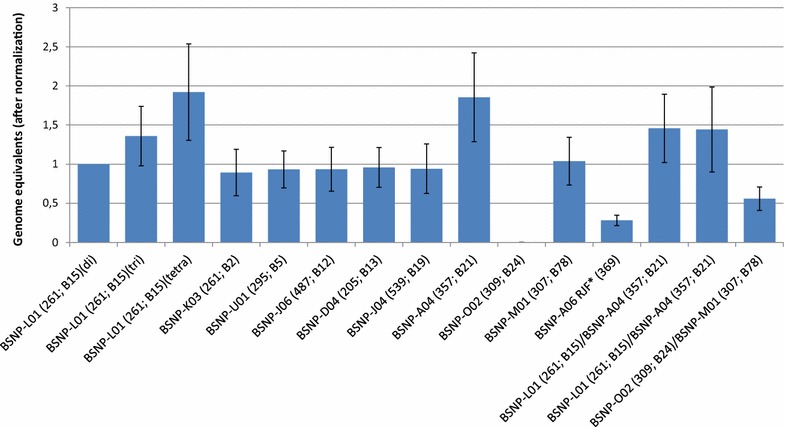
qPCR results for SNP MHCNew10. DNA samples tested included MHC-*B15* disomic, trisomic and tetrasomic samples, followed by multiple MHC-*B* homozygotes (all disomic) and defined MHC-*B* heterozygotes. Results were all normalized to *B15* disomic DNA and an internal single-copy gene control *RNAseP*

These putative genome copy number variants were further investigated by qPCR and the results are summarized in Fig. 5. Data are presented as genome equivalents after normalization to an internal single copy number gene (RNAseP) and normalization to one of the disomic samples [BSNP-L01 (261; B15)]. All tested samples had known MHC-*B* haplotypes as indicated on the graph. The disomic, trisomic and tetrasomic samples are from individuals known to have two, three or four copies of the MHC, respectively, and all were homozygous for *B15* [1]. The graph shows a clear step-wise progression from 1 to 1.5 and then 2-genome equivalents as expected for aneuploid samples. The next samples are examples of nine different haplotypes. These show 1-genome equivalent for the six haplotypes [BSNP-K03 (261; B2, B29), BSNP-U01 (295; B5), BSNP-J06 (461, 474, 487; B71, B12, B12.3), BSNP-D04 (205; B13) BSNP-J04 (539, 552; B19, B19.1), and BSNP-M01 (295, 307, 319; B72, B78)], 2-genome equivalents for haplotype BSNP-A04(357; B21) and no amplification for haplotype BSNP-O02 (309; B24). These results are consistent with the interpretation of the results in Fig. 4, i.e., BSNP-A04 (357; B21) haplotype has 2-genome equivalents and haplotype BSNP-O02 (309; B24) has no genome equivalent for this region. The two individuals that were heterozygous for BSNP-L01 (261; B15)/BSNP-A04 (357; B21), both show 1.5-genome equivalents as predicted for an individual heterozygous for 1-genome and 2-genome equivalent haplotypes. The individual heterozygous for BSNP-O02 (309; B24)/BSNP-M01 (295, 307, 319; B72, B78), shows a 0.5-genome equivalent as predicted for this haplotype heterozygous combination (i.e. a no copy and single copy combination). The RJF reference sample is considered to be homozygous for the MHC and consistently gave an unexpected result, which indicates that less than half a genome equivalent is found in repeated assays. The amplification efficiency of the qPCR was lower for the RJF reference DNA than for any other sample, which suggests that sequence variation at PCR binding sites may contribute to this aberrant result.

The six SNPs (MHCNew07 to MHCNew12) are located between positions 12,062 and 13,992 bp and are all within the *BG2* gene. The unusual SNP genotype results observed for this group of SNPs is consistent with either deletion or duplication events in this particular gene, depending on the haplotype.

Additional apparent gene duplications (as indicated by apparent SNP heterozygosity in some samples that are homozygous for the rest of the haplotype) were also observed for other SNPs in this region. MHCNew17 (in exon of *KIFC1*, 21,784 bp) appeared to be heterozygous for samples that were homozygous for 11 haplotypes and MHCNew19 (in exon of *LOC425771*, 27,791 bp) appeared to be heterozygous for samples that were homozygous for five haplotypes, which suggests that other genes or parts of gene in this region are duplicated in some haplotypes. No evidence of deletion of either of these two genes was found. DNA from the RJF reference genome [BSNP-B06 (369) RJF*] contained both SNP alleles (and thus potentially contained a duplicated priming site for SNPs MHCNew17, MHCNew19, MHCNew20 and MHCNew21).

Overall, the SNPs that encompassed positions 9000 to 27,000 bp revealed interesting characteristics. For this region, the end-point read results of the KASP assay suggested the occurrence of gene duplication and deletion events in the different haplotypes for the *BG2* gene. The qPCR results for SNP MHCNew10 confirmed the duplication in one haplotype (BSNP-A04 (357; B21) and deletion in another (BSNP-O02 (309; B24).

This endpoint read PCR method provided rapid preliminary evidence of gene duplication and deletion. The results highlighted other haplotypes that should be investigated more thoroughly in future studies. The qPCR results of the RJF reference DNA are intriguing i.e. PCR efficiency of the reference sample was lower than that of all tested samples, which suggested the presence of additional unknown variation within the region that is unique to the reference genome haplotype. The sequence of the RJF reference DNA showed the presence of other SNPs with apparent duplications which suggests that additional variation may occur in other genes within this sequence. Multiple sections of highly similar sequences may well explain some of the difficulties that were encountered with sequence alignment of the MHC reference. Further efforts are needed to better understand and catalog the duplication and deletion of genes in this region for multiple haplotypes. From these observations, we conclude that the genes within the first 27,000 bp of the GenBank: AB268588 region are subject to gene duplications and deletions. These findings are consistent with the observation of Salomonsen et al. that recombination and/or deletion events are likely major evolutionary forces that shaped this portion of MHC-*B* [82].

### LEI0258 variation and mutations

To complete the picture of the variability in the MHC-*B* region, we included LEI0258 in the BSNP haplotypes (see Additional file 2: Table S2). Close examination of the SNP-defined haplotypes revealed distinct haplotypes that shared the same LEI0258 allele. For example, the serologically distinct well-defined haplotypes of *B2* [BSNP-K03 (261; B2, B29)] and *B15* [BSNP-L01 (261; B15)] share the same 261-bp LEI0258 allele, yet they differ at 31 SNPs across the entire region. Similarly, other SNP-defined (and serologically-defined) haplotypes that are different can share LEI0258 alleles. These include *B13* [BSNP-D04 (205; B13)] and *B17* [BSNP-N01 (205; B17)], which share a 205-bp LEI0258 allele but differ by 41 SNPs, and *B21* [BSNP-A04 (357; B21)] and *B23* [BSNP-I01 (357; B23)] that share a 357-bp LEI0258 allele but differ by 43 SNPs. In this study, we found that the 357-bp LEI0258 allele was shared by nine SNP haplotypes.

Conversely, some identical SNP-defined haplotypes differed at LEI0258. For example, haplotype BSNP-J06 (461, 474, 487; B71, B12, B12.3) had three different LEI0258 alleles (461, 474 or 487 bp). Similarly, BSNP-M01 (295, 307, 319; B72, B78) had three LEI0258 alleles. In other cases, two different LEI0258 alleles were found for haplotypes BSNP-A09A (357, 369; BQ), BSNP-A08 (357, 369; B77), BSNP-J04(539, 552; B19, B19.1), and BSNP-V07 (393, 405; B1, B1.2). Each of the LEI0258 alleles found with the same BSNP haplotype differ by either 12 or 13 bp, suggesting that they are due to mutation of one of the LEI0258 repeats.

For the samples tested herein, 29 different LEI0258 alleles were identified and ranged from 182 to 552 bp in length (LEI0258 alleles generally differ by multiples of 12 or 13 bp). Typing of three commercial lines for which pedigree information was available revealed three LEI0258 allele size mutations (all corresponding to 12-bp increases from the parental allele) among 2667 birds. This is equivalent to a LEI0258 mutation rate of 1.1/1000 which is lower than the 6/1000 MHC-*B* crossover rate observed in this study.

## Conclusions

A relatively simple and scalable SNP-typing method was developed that allows new MHC-*B* haplotypes to be detected regardless of whether they were previously serologically-defined haplotypes that existed in the populations, haplotypes with no serological information or new haplotypes that originated from recombination, which can occur at many points within the MHC-*B* region. We observed recombination rates that are 10-fold higher than previously reported using serological detection, which is likely due to improved detection efficiency using the SNP panel. While the number of genes in the 3′-portion of the region is relatively stable, genes at the 5′-end are apparently subject to copy number variation. MHC-*B* haplotype diversity is often under-represented by the linked VNTR LEI0258. Furthermore, the mutation rate of LEI0258 is a source of error in estimating MHC variation with this marker only. It should be noted that this SNP panel defined haplotypes based only on variation at the reported SNPs. Additional variation may occur at other sites in the intervening sequences that are not interrogated by the reported SNPs. Reliable detection of SNPs within highly variable genes such as *BF* and *BLB* can be problematic since multiple SNPs that lie in close proximity (i.e., within the length of the oligonucleotide primer) can inhibit primer binding. Identification of this additional variation would require additional SNPs and perhaps in-depth sequencing. Considering the strong association between MHC-*B* and disease resistance and the increasing concern of emerging avian diseases world-wide, a reliable and consistent method to identify MHC-*B* variability in chicken populations, including the wild Jungle fowl ancestors is necessary. Furthermore, examination of MHC-*B* haplotypes in ancestral populations should help to better understand the evolution of the MHC within the context of domestication.

## Additional files

10.1186/s12711-015-0181-x SNPs used for the MHC panel. Genes and bp locations are provided based on the sequence [GenBank: AB268588] as well as the primer sequences that were used to develop the KASP-based detection assay.
10.1186/s12711-015-0181-x Detected MHC SNP-based haplotypes. MHC SNP-based information for all haplotypes detected for the serologically-defined lines, commercial elite layer stocks and heritage breeds.
10.1186/s12711-015-0181-x Detected MHC SNP-based recombinant haplotypes. Information includes the parental haplotypes and the range (defined by SNPs) within which each recombination occurred.

## References

[1] Miller MM, Goto RM, Taylor RL, Zoorob R, Auffray C, Briles RW (1996). Assignment of Rfp-Y to the chicken major histocompatibility complex/NOR microchromosome and evidence for high-frequency recombination associated with the nucleolar organizer region. Proc Natl Acad Sci USA.

[2] Schierman LW, Nordskog AW (1961). Relationship of blood type to histocompatibility in chickens. Science.

[3] Hofmann A, Plachy J, Hunt L, Kaufman J, Hala K (2003). v-src oncogene-specific carboxy-terminal peptide is immunoprotective against Rous sarcoma growth in chickens with MHC class I allele B-F12. Vaccine..

[4] Goto RM, Wang Y, Taylor RL, Wakenell PS, Hosomichi K, Shiina T (2009). BG1 has a major role in MHC-linked resistance to malignant lymphoma in the chicken. Proc Natl Acad Sci USA.

[5] Miller MM, Taylor JR (2016). Brief review of the chicken major histocompatibility complex—the genes, their distribution on chromosome 16 and their contributions to disease resistance. Poult Sci.

[6] Cotter PF, Taylor RL, Abplanalp H (1998). B-complex associated immunity to Salmonella enteritidis challenge in congenic chickens. Poult Sci.

[7] Lamont SJ, Bolin C, Cheville N (1987). Genetic resistance to fowl cholera is linked to the major histocompatibility complex. Immunogenetics.

[8] Hansen MP, Van Zandt JN, Law GRJ (1967). Differences in susceptibility to Marek’s disease in chickens carrying two different B locus blood group alleles. Poult Sci.

[9] Briles W, Stone H, Cole R (1977). Marek’s disease: effects of B histocompatibility alloalleles in resistant and susceptible chicken lines. Science.

[10] Bacon LD, Hunt HD, Cheng HH (2001). Genetic resistance to Marek’s disease. Curr Top Microbiol Immunol.

[11] Bacon LD, Witter RL, Crittenden LB, Fadly A, Motta J (1981). B-haplotype influence on Marek’s disease, Rous sarcoma, and lymphoid leukosis virus-induced tumors in chickens. Poult Sci.

[12] Taylor RL (2004). Major histocompatibility (B) complex control of responses against Rous sarcomas. Poult Sci.

[13] Yoo BH, Sheldon BL (1992). Association of the major histocompatibility complex with avian leukosis virus infection in chickens. Br Poult Sci.

[14] Owen JP, Delany ME, Mullens BA (2008). MHC haplotype involvement in avian resistance to an ectoparasite. Immunogenetics.

[15] Worley K, Collet J, Spurgin LG, Cornwallis C, Pizzari T, Richardson DS (2010). MHC heterozygosity and survival in red junglefowl. Mol Ecol.

[16] Lillehoj HS, Lillehoj EP, Weinstock D, Schat KA (1988). Functional and biochemical characterizations of avian T lymphocyte antigens identified by monoclonal antibodies. Eur J Immunol.

[17] Schou TW, Labouriau R, Permin A, Christensen JP, Sørensen P, Cu HP (2010). MHC haplotype and susceptibility to experimental infections (*Salmonella Enteritidis*, *Pasteurella multocida* or *Ascaridia galli*) in a commercial and an indigenous chicken breed. Vet Immunol Immunopathol.

[18] Lee LF, Bacon LD, Yoshida S, Yanagida N, Zhang HM, Witter RL (2004). The efficacy of recombinant fowlpox vaccine protection against Marek’s disease: its dependence on chicken line and B haplotype. Avian Dis.

[19] Pinard-Van der Laan MH, Siegel PB, Lamont SJ (1998). Lessons from selection experiments on immune response in the chicken. Poult Avian Biol Rev..

[20] Lamont SJ (1998). Impact of genetics on disease resistance. Poultry Sci..

[21] Juul-Madsen HR, Dalgaard TS, Rontved CM, Jensen KH, Bumstead N (2006). Immune response to a killed infectious bursal disease virus vaccine in inbred chicken lines with different major histocompatibility complex haplotypes. Poult Sci.

[22] Lamont SJ (1998). The chicken major histocompatibility complex and disease. Rev Sci Tech OIE..

[23] Bacon LD, Hunt HD, Cheng HH (2000). A review of the development of chicken lines to resolve genes determining resistance to diseases. Poult Sci.

[24] Abplanalp H, Sato K, Napolitano D, Reid J (1992). Reproductive performance of inbred congenic Leghorns carrying different haplotypes for the major histocompatibility complex. Poult Sci.

[25] Cotter PF, Taylor RL, Abplanalp H (1992). Differential resistance to *Staphylococcus aureus* challenge in major histocompatibility (B) complex congenic lines. Poult Sci.

[26] Plachy J, Jurajda V, Benda V (1984). Resistance to Marek’s disease is controlled by a gene within the B-F region of the chicken major histocompatibility complex in Rous sarcoma regressor or progressor inbred lines of chickens. Folia Biol (Praha).

[27] Plachy J, Vilhelmova M, Karakoz I, Schulmannova J (1989). Prague inbred lines of chickens: a biological model for MHC research. Folia Biol.

[28] Valdez MB, Mizutani M, Fujiwara A, Yazawa H, Yamagata T, Shimada K (2007). Histocompatible chicken inbred lines: homogeneities in the major histocompatibility complex antigens of the GSP, GSN/1, PNP/DO and BM-C inbred lines assessed by hemagglutination, mixed lymphocyte reaction and skin transplantation. Exp Anim.

[29] Briles WE, Bumstead N, Ewert DL, Gilmour DG, Gogusev J, Hála K (1982). Nomenclature for chicken major histocompatibility (B) complex. Immunogenetics.

[30] Fulton JE, Thacker EL, Bacon LD, Hunt HD (1995). Functional analysis of avian class I (BFIV) glycoproteins by epitope tagging and mutagenesis in vitro. Eur J Immunol.

[31] Miller MM, Bacon LD, Hala K, Hunt HD, Ewald SJ, Kaufman J (2004). 2004 Nomenclature for the chicken major histocompatibility (B and Y) complex. Immunogenetics.

[32] Hosomichi K, Miller MM, Goto RM, Wang Y, Suzuki S, Kulski JK (2008). Contribution of mutation, recombination, and gene conversion to chicken MHC-B haplotype diversity. J Immunol..

[33] Longenecker BM, Gallatin WM. Genetic control of resistance to Marek’s disease. IARC Sci Publ. 1978; (24 Pt 2):845–50. 109390

[34] Schou M, Crone M, Simonsen M (1982). The major histocompatibility complex of outbred chickens. I. Analysis of the B13 haplotype by serology and cellular reactions. Tissue Antigens.

[35] Miller MM, Abplanalp H, Goto R (1988). Genotyping chickens for the B-G subregion of the major histocompatibility complex using restriction fragment length polymorphisms. Immunogenetics.

[36] Hala K, Sgonc R, Auffray C, Wick G (1989). Typing of MHC haplotypes in OS chicken by means of RFLP analysis. Prog Clin Biol Res.

[37] Chaussé AM, Coudert F, Dambrine G, Auffray C (1989). Analysis of five B complex haplotypes with class I (B-F), class II (B- L) and class IV (B-G) probes. Prog Clin Biol Res.

[38] Pinard MH, Hepkema BG (1993). Biochemical and serological identification of major histocompatibility complex antigens in outbred chickens. Vet Immunol Immunopathol.

[39] Goto R, Briles WE, Miller MM (1994). RFLP differences identified in sequential chicken B recombinant haplotypes derived from B^23^. Anim Genet.

[40] Zheng D, O’Keefe G, Li L, Johnson LW, Ewald SJ (1999). A PCR method for typing B-L beta II family (class II MHC) alleles in broiler chickens. Anim Genet.

[41] Nishibori M, Nakaki S, Tsudzuki M, Yamamoto Y (2000). Utility of three restriction fragment length polymorphism probes for genotyping of the chicken major histocompatibility complex class IV region. Poult Sci.

[42] Livant EJ, Zheng D, Johnson LW, Shi W, Ewald SJ (2001). Three new MHC haplotypes in broiler breeder chickens. Anim Genet.

[43] Goto RM, Afanassieff M, Ha J, Iglesias GM, Ewald SJ, Briles WE (2002). Single-strand conformation polymorphism (SSCP) assays for major histocompatibility complex B genotyping in chickens. Poult Sci.

[44] Iglesias GM, Soria LA, Goto RM, Jar AM, Miquel MC, Lopez OJ (2003). Genotypic variability at the major histocompatibility complex (B and Rfp-Y) in Camperos broiler chickens. Anim Genet.

[45] Livant EJ, Ewald SJ (2005). High-resolution typing for chicken BF2 (MHC class I) alleles by automated sequencing. Anim Genet.

[46] Fulton JE, Juul-Madsen HR, Ashwell CM, McCarron AM, Arthur JA, O’Sullivan NP (2006). Molecular genotype identification of the *Gallus gallus* major histocompatibility complex. Immunogenetics.

[47] Chazara O, Juul-Madsen HR, Chang CS, Tixier-Boichard M, Bed’hom B. Correlation in chicken between the marker LEI0258 alleles and major histocompatibility complex sequences. BMC Proc. 2011; 5 S29. 10.1186/1753-6561-5-S4-S29 PMC3108224 21645309

[48] Chazara O, Tixier-Boichard M, Morin V, Zoorob R, Bed’hom B (2011). Organisation and diversity of the class II DM region of the chicken MHC. Mol Immunol.

[49] Chazara O, Pinard-van Der Laan MH, Tixier-Boichard M, Bed’hom B (2008). Molecular genotype investigation of the *Gallus gallus* major histocompatibility complex. Dev Biol..

[50] Chang CS, Chen CF, Berthouly-Salazar C, Chazara O, Lee YP, Chang CM (2012). A global analysis of molecular markers and phenotypic traits in local chicken breeds in Taiwan. Anim Genet.

[51] Chazara O, Chang CS, Bruneau N, Benabdeljelil K, Fotsa JC, Kayang BB (2013). Diversity and evolution of the highly polymorphic tandem repeat LEI0258 in the chicken MHC-B region. Immunogenetics.

[52] McConnell S, Dawson D, Wardle A, Burke T (1999). The isolation and mapping of 19 tetranucleotide microsatellite markers in the chicken. Anim Genet.

[53] Nikbakht G, Esmailnejad A (2015). Chicken major histocompatibility complex polymorphism and its association with production traits. Immunogenetics.

[54] Guangxin E, Sha R, Zeng S, Wang C, Pan J, Han J (2014). Genetic variability, evidence of potential recombinational event and selection of LEI0258 in chicken. Gene..

[55] Han B, Lian L, Qu L, Zheng J, Yang N (2013). Abundant polymorphisms at the microsatellite locus LEI0258 in indigenous chickens. Poult Sci.

[56] Nikbakht G, Esmailnejad A, Barjesteh N (2013). LEI0258 microsatellite variability in Khorasan, Marandi, and Arian chickens. Biochem Genet.

[57] Izadi F, Ritland C, Cheng KM (2011). Genetic diversity of the major histocompatibility complex region in commercial and noncommercial chicken flocks using the LEI0258 microsatellite marker. Poult Sci.

[58] Suzuki K, Matsumoto T, Kobayashi E, Uenishi H, Churkina I, Plastow G (2010). Genotypes of chicken major histocompatibility complex B locus associated with regression of Rous sarcoma virus J-strain tumors. Poult Sci.

[59] Bader SR, Kothlow S, Trapp S, Schwarz SC, Philipp HC, Weigend S (2010). Acute paretic syndrome in juvenile White Leghorn chickens resembles late stages of acute inflammatory demyelinating polyneuropathies in humans. J Neuroinflammation..

[60] Schou TW, Permin A, Juul-Madsen HR, Sørensen P, Labouriau R, Nguyên TL (2007). Gastrointestinal helminths in indigenous and exotic chickens in Vietnam: association of the intensity of infection with the Major Histocompatibility Complex. Parasitology.

[61] Briles WE, Briles RW. Genetic recombination within the B blood group system of chickens. Proceedings of the Conference of the International Society for Animal Blood Group Research: Dublin. 1976. pp. 8.

[62] Briles WE, Briles RW, Benedict AA (1977). Some recent recombinants at the B locus. Avian immunology—advances in experimental medicine and biology.

[63] Briles WE, Briles RW (1980). A search for “duplicate” recombinants between B-F and B-G regions in the chicken B complex. Anim Blood Groups Biochem Genet..

[64] Briles WE, Briles RW, Taffs RE (1982). An apparent recombinant within the B-G region of the B complex. Poult Sci.

[65] Simonsen M, Hala K, Nicolaisen EM (1980). Linkage disequilibrium of MHC genes in the chicken. I. The B-F and B-G loci. Immunogenetics.

[66] Pevzner IY, Trowbridge CL, Nordskog AW (1978). Recombination between genes coding for immune response and the serologically determined antigens in the chicken B system. Immunogenetics.

[67] Koch C, Skjodt K, Toivanen A, Toivanen P (1983). New recombinants within the MHC (B-complex) of the chicken. Tissue Antigens.

[68] Hunt HD, Pharr GT, Bacon LD (1994). Molecular analysis reveals MHC class I intra-locus recombination in the chicken. Immunogenetics.

[69] Shiina T, Briles WE, Goto RM, Hosomichi K, Yanagiya K, Shimizu S (2007). Extended gene map reveals tripartite motif, C-type lectin, and Ig superfamily type genes within a subregion of the chicken MHC-B affecting infectious disease. J Immunol..

[70] Fulton JE, Lund AR, McCarron AM, Korver DR, Classen HL, Aggrey S (2016). MHC Variability in Heritage breeds of chickens. Poult Sci.

[71] Chazara O. Diversite genetique structurale et fonctionnelle du CMH chezle poult: implication opur la resitance aux maladies. PhD thesis, AgroParisTech. 2010.

[72] Semagn K, Beyene Y, Warburton ML, Tarekegne A, Mugo S, Meisel B (2013). Meta-analyses of QTL for grain yield and anthesis silking interval in 18 maize populations evaluated under water-stressed and well-watered environments. BMC Genomics.

[73] Kranis A, Gheyas AA, Boschiero C, Turner F, Yu L, Smith S, Talbot R (2013). Development of a high density 600K SNP genotyping array for chicken. BMC Genomics.

[74] Paradis E, Claude J, Strimmer K (2004). APE: analyses of phylogenetics and evolution in R language. Bioinformatics.

[75] Saitou N, Nei M (1987). The neighbor-joining method: a new method for reconstructing phylogenetic trees. Mol Biol Evol.

[76] Margush T, McMorris FR (1981). Consensus n-trees. Bull Math Biol.

[77] Felsenstein J (1985). Confidence limits on phylogenies: an approach using the bootstrap. Evolution.

[78] Prosperi MC, Ciccozzi M, Fanti I, Saladini F, Pecorari M, Borghi V (2011). A novel methodology for large-scale phylogeny partition. Nat Commun..

[79] Bloom SE, Bacon LD (1985). Linkage of the major histocompatibility (B) complex and the nucleolar organizer in the chicken. Assignment to a microchromosome. J Hered..

[80] Nguyen-Phuc H, Fulton JE and Berres ME. Genetic variation of Major Histocompatibility Complex (MHC) in wild Red JungleFowl (*Gallus gallus*). Poultry Sci. 2016 (in press). 10.3382/ps/pev364 26839415

[81] Briles WE, Briles RW (1982). Identification of haplotypes of the chicken major histocompatibility complex (B). Immunogenetics.

[82] Salomonsen J, Chattaway JA, Chan AC, Parker A, Huguet S, Marston DA (2014). Sequence of a complete chicken BG haplotype shows dynamic expansion and contraction of two gene lineages with particular expression patterns. PLoS Genet.

